# The signalling game between plants and pollinators

**DOI:** 10.1038/s41598-018-24779-0

**Published:** 2018-04-27

**Authors:** Shan Sun, Michael I. Leshowitz, Jan Rychtář

**Affiliations:** 1 State Key Laboratory of Grassland Agro-Ecosystems, School of Life Sciences, Lanzhou University, Lanzhou, 730000 People’s Republic of China; 2 Department of Mathematics and Statistics, The University of North Carolina at Greensboro, Greensboro, NC USA

## Abstract

Plants can send floral signals to advertise their reward for pollinators. Based on the presence or absents of such signals, pollinators can determine whether to visit plants. Plants can send dishonest signals but foraging behaviours of pollinators can limit the cheating strategies of plants. We model the plant-pollinator interactions by the two-type Spence signalling game and investigate the conditions under which honest signalling can be established. In our model, plants either send costly signal or they do not. The cost of signal is dependent on the quality of plant. Pollinators can learn from the interactions with plants and can update their willingness to visit plants’ flowers to maximize their foraging efficiency. We find three general conditions that are required for the evolutionary stability of honest signaling. Those conditions are satisfied if there is (a) a high frequency of high-yield signalling plants in the population, (b) the balance between cost and benefit of signalling, and (c) high cost of dishonest signalling. Our model also predicts that other factors contributing to the establishment of honest signaling are the low abundance of pollinators, and the positive density-dependent and positive frequency-dependent relationship between plants and pollinators.

## Introduction

The cooperation between plants and their pollinators could develop through the exchange of rewards and services. One of main problems deterring the establishment of cooperation between species is the existence hidden characteristics due to information asymmetry^1^: the plants know their own quality and can therefore behave accordingly, but the pollinators do not a priori know the quality of the plants. Specifically, plants often conceal the reward (e.g. nectar and/or pollen) within the flower and pollinators cannot directly detect it. This type of pollinator-plant interactions with information asymmetry could be understood as a problem of “partner choice”^1,2^. Signalling could provide a mechanism to solve this problem^1^. The plants could signal their reward quality or quantity, and the pollinators could use the plants’ signals to determine whether or not to visit the plants.

In signalling game between plants and pollinators, honest signals are those that are positively correlated with the amount of rewards. The most honest signalling is the direct and conspicuous display of the reward itself ^3^. For example, the scent of pollen in *Rosa rugosa*^4^ and nectar in *Penstemon caesius*^5^, coloured nectar^6^, and the size of the visible gland secreting reward in *Dalechampia schottii*^3^ could be as visual and/or olfactory signals to the pollinators. The floral traits that are uncoupled from the rewards within flowers could be as honest signals to advertise rewards for pollinators. The floral signals positively correlated with the amount of rewards have been documented for floral colour changes after post-pollination^7,8^, the size of petals and corolla-tube diameters in *Silene virginica*^9^, corolla tube length in *Erysimum mediohispanicum*^10^, petal length in *Turnera ulmifolia*^11^, symmetrical flowers in *Epilobium angustifolium*^12^, bract size in *Dalechampia ipomoeifolia*^13^, and floral scent in *Brassica rapa*^14^.

In plant-pollinator signalling game, the central question is how the honest signals have been established when the interests of signallers and receivers conflict partly. On the one hand, because floral signals and rewards are uncoupled within one flower, plants could reduce the cost by sending dishonest signals (signals with low correlation to the amount of rewards)^15^. According to costly signaling theory^16–18^, the cost of signals is an essential element for stable honest signaling in any signalling scenario and it thus extends to the plant-pollinator interactions. If the benefits for low and high quality signallers are same, the effectiveness of signalling depends on the strength of the correlation between the cost and the quality desired by the receiver: the cost must be higher for the low-quality sender^1,17,19^. When the signal has a cost, only good quality individuals will find it profitable to advertise their quality, therefore the signal will be honest. On the other hand, pollinators usually rely on floral signals to assess the amount of rewards. The foraging behaviour of pollinators could lead to the honest signals offered by plants though repeated interactions with plants. Pollinators, such as bumble bees, not only learn positive or adverse associations between floral signals and rewards^20^, but also gather the information about reward amount and use this information to improve their subsequent foraging efficiency in repeated interactions with plants^21–23^. The pollinators could remember the most profitable patches, and return preferentially to these^21^. The experienced pollinators, *Bombus ignitus*, could discriminate between rewarding and less-rewarding plants and return more frequently to plants providing high amounts of reward^22^. *Bombus impatiens* follow a Bayesian updating process to assess the degree of correlation between flower signals and rewards^23^. When pollinators could update the information about signal-reward relationship based on past experiences, the foraging behaviour of pollinators could limit the dishonest signals in by a preference for honest ones correlated to rewards in *T. ulmifolia*^11^. The hawkmoth pollinators, *Manduca sexta*, can make the adjustment of probing time in response to nectar volume, and that the self-serving pollinator behaviour can reduce probing duration and in low-nectar plants, resulting in reduced seed production^24^. Broom *et al*. (2013) have demonstrated theoretically that signal verification as self-serving strategy, which the receiver is able to verify the accuracy of the signal after it has responded to the signal, can lead to signal reliability by reducing interactions with dishonest signalers in repeated interactions^25^. The interaction frequency could determine the effect of the foraging behaviour of pollinators on plants.

However, it is still not clear whether the cost of signals combined with foraging behaviour of pollinators could make the honest signaling to be a stable equilibrium in the repeated plant-pollinator signalling game. In this study, we investigated theoretically the establishment of honest signals in repeated two-type Spence signalling game between plants and pollinators. The Spence signalling game captures the central feature of signalling game that strategic costs associated with signalling are required for honest signalling in the presence of partial conflict of interest^19^.

The Spence signalling game belongs to the family of the differential cost games (such as Zahavi^16^ and Grafen^17^), in which one type pays lower signalling costs than the other while both types gain the same benefits. The game is similar but not identical to the Sir Phillip-Sydney game^18^ which inspired a lot of models that investigate the conditions of honest signalling (see for example Hurd^26^; Bullock^27^; Számadó^28^). Unlike the Spence signalling game, the Sir Phillip-Sydney game belongs to the differential benefit models, in which one type reaps a larger reward from the receiver’s response than the other, while the cost of signalling is same for each type of sender.

In our repeated signalling game, we assume that plants with different quality have different signalling cost and that they also provide different benefit for pollinators. In each visiting bout, the plants could either send the signal or not. Potentially based on the signal, the pollinator determines the probability to visit the plant. The probability of pollinator visitation could be updated based on the consequences for plant-pollinator interaction. Under this situation, we would explore whether the honest signalling can be established as evolutionarily stable strategy in the repeated game. Our model could provide the framework for using signalling game combined with pollinators’ foraging strategies to resolve the establishment of mutualism between plants and pollinators.

## Model

For the sake of the simplicity, we assume that there are only two kinds of plants: (1) high-yield plants that provide benefit *V*_*H*_ to the pollinators and (2) low-yield plants that provide benefit *V*_*L*_ < *V*_*H*_ to the pollinators. Let the relative frequency of high-yield plants be 0 < *h* < 1. Both kinds of plants can send signals to pollinators, advertising (potentially dishonestly) a high reward. A strategy for a plant is thus to signal or not (and it potentially depends on whether the plant is high-yield or low-yield). The cost of the signalling for a high-yield plant is *C*_*H*_, the cost of a signal for low-yield plant is assumed to be *C*_*L*_. The benefits of pollination are same for both the high-yield as well as the low-yield plants. We will later see that for an honest signalling (i.e. only high-yield plans signal) to develop in this situation, we need *C*_*L*_ > *C*_*H*_ (which is part of the Spence condition for honest signalling, see e.g., Fudenberg and Tirole^29^).

When a pollinator finds a plant, it checks for signals. If the plant is signaling, the pollinator visits the flowers with probability *v*_*s*_. If the plant is not signaling, the pollinator visits it with probability *v*_*N*_. We assume the visit takes the pollinator time *T*_*V*_. If the pollinator decides not to visit, it will take additional time *T*_*F*_ to find another plant.

We summarize the notation in Table 1.

**Table 1:** Explanation of used symbols and notation.

Symbol	Meaning
*V*_*H*_	Benefits to pollinators provided by high-yield plants
*V*_*L*_	Benefits to pollinators provided by low-yield plants, *V*_*L*_ < *V*_*H*_
*h*	Relative frequency of high-yield plants, 0 < *h* < 1
*C*_*H*_	The cost of the signalling for a high-yield plant
*C*_*L*_	The cost of the signalling for a low-yield plant, *C*_*L*_ > *C*_*H*_
*v*_*S*_	Probability a pollinator visits a signalling plant
*v*_*N*_	Probability a pollinator visits a non-signalling plant
*v*_*max*_	Maximal probability to visit a plant (signalling or not)
*v*_*min*_	Minimal probability to visit a plant (signalling or not), $0\le {v}_{min} < {v}_{max}\le 1$
*T*_*V*_	Time it takes pollinator to visit a plant
*T*_*F*_	Time it takes pollinator to find another plant
*T*	Expected time to receive reward from a plant (if pollinator visits only signalling high-yield plants)
*R*(*v*)	Reward for the plant that is visited by pollinators with probability *v*
*R*_0_	Base reward for a plant when a pollinator visits one of its flowers
${R}_{single}(v)$	An example of a reward for a plant with a single flower, ${R}_{single}(v)={R}_{0}(1-{(1-v)}^{P})$
${R}_{multi}(v)$	An example of a reward for a plant with multiple flower ${R}_{multi}(v)={R}_{0}vP$
*P*	Number of pollinators that can visit a plant

Whenever a pollinator visits a plant, it updates the visiting probabilities accordingly. To keep our model as general as possible, we will not assume any specific updating rules (see for example Cartar^21^; Makino and Sakai^22^; Biernaskie *et al*.^23^). However, we will assume that pollinators try to maximize their benefits based on optimal foraging theory^30^, i.e. the amount of food/rewards collected per unit of time. Since the high-yield plant provides the best reward possible, visiting a high-yield plant will thus make visits of plants doing the same more likely (whether the plant signaled or not). Reinforcement learning algorithms (see for example Sutton & Barto^31^) satisfy this requirement. We will explore the learning outcome of visit of low-yield plant in the next section.

The time lag between the response for the signal and its fitness returns can affect the learning that is the ability to update the template of the signal-reward relationship^32^. We will also assume that pollinators have sufficiently long time to learn, i.e. the values of *v*_*s*_ and *v*_*N*_ have converged to their expected (and fixed) values. Different learning rules may yield different results but we will assume that the expected values are between 0 ≤ *v*_*min*_ < *v*_*max*_ ≤ 1.

**Figure 1:**
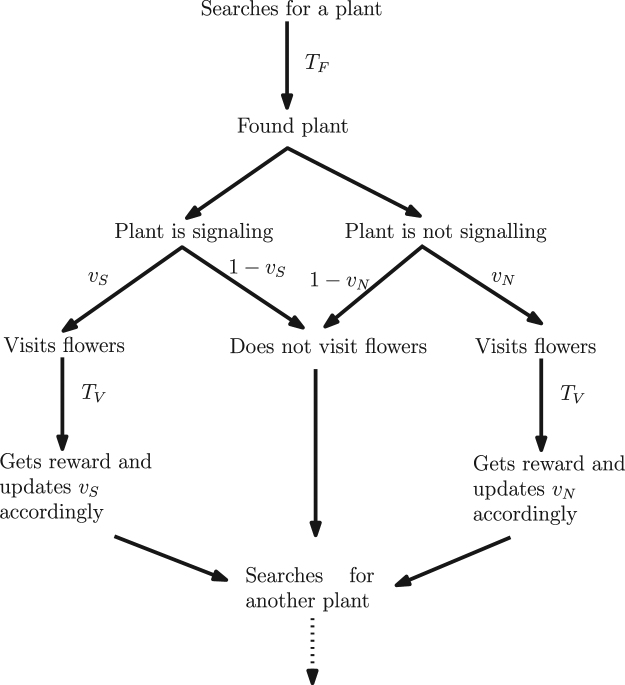
The game from the pollinator’s perspective. On average, it takes time *T*_*F*_ to find a plant. Once the pollinator finds a plant, it visits it with probability *v*_*S*_ (for the signalling plant) or *v*_*N*_ (for the non-signalling plant), respectively. The pollinator spends time *T*_*v*_ visiting either type of the plant. Whenever the pollinator visits a plant, it gets a reward *V*_*H*_ if it was a high-yield plant, or it gets a reward *V*_*L*_ < *V*_*H*_ if it was a low yield plant. The rewards for the plants depend on how likely they are visited by the pollinators and the reward functions are discussed in the text.

The game from the pollinator’s perspective is shown in Fig. 1.

The rewards for the plants will depend on how frequently their flowers are visited by pollinators. To be as general as possible, we will just assume that if a plant is visited by pollinators with probability *v*, the reward for the plant will be *R*(*v*) for some function *R* that is increasing in *v*. If the plant does not signal, the total net benefit for the plant will simply be *R*(*v*_*N*_) as there is no signaling cost. If the plant signals, the total net benefit will be *R*(*v*_*S*_) − *C* where is the cost of the signaling and it is either *C*_*H*_ or *C*_*L*_, depending on whether the plant is high-yield or low-yield.

Just for the illustration, here are some possible reward functions. If a plant has just one flower and it can potentially be visited by *P* pollinators, the potential reward function may be given by ${R}_{single}(v)={R}_{0}(1-{(1-v)}^{P})$ corresponding to the fact that the plant’s flower is visited with probability $(1-{(1-v)}^{P})$ and the plant receives a base reward *R*_0_ when a pollinator visits the flower. Alternatively, when the plant has many flowers (significantly more than *P*), and each pollinator visits only one flower, the reward for the plant is given by ${R}_{multi}(v)={R}_{0}vP$. Clearly, there are many more possibilities for reward functions^33,34^. While both functions ${R}_{single}(v)$ and ${R}_{multi}(v)\,$are increasing in *v*, there are some significant differences between the two functions. For example, for large *P*, i.e. when pollinators are abundant, ${R}_{single}(v)\approx {R}_{0}$ is essentially constant while *R*_*multi*_ is still linear with a constant slope.

## Results

Here we show results of the ESS analysis. We assume that all (but a negligible fraction) of plants use a given strategy and we derive conditions under which such strategy cannot be successfully invaded by negligible fraction of plants using a different strategy. Pollinators learn based on the experience with the plants they encounter and since we assume that potential invaders come in negligible fraction, the invaders will not influence the learning of pollinators at all.

For the majority of this section, we will assume that essentially all high-yield plants are signaling and essentially all low-yield plants do not. In the following section, we will determine the long-term pollinator feeding rate, specify conditions under which pollinators should learn to skip low-yield non-signaling plants and finally determine all conditions under which honest signaling is evolutionarily stable strategy for the plants.

### Long-term pollinator feeding rate

**Figure 2:**
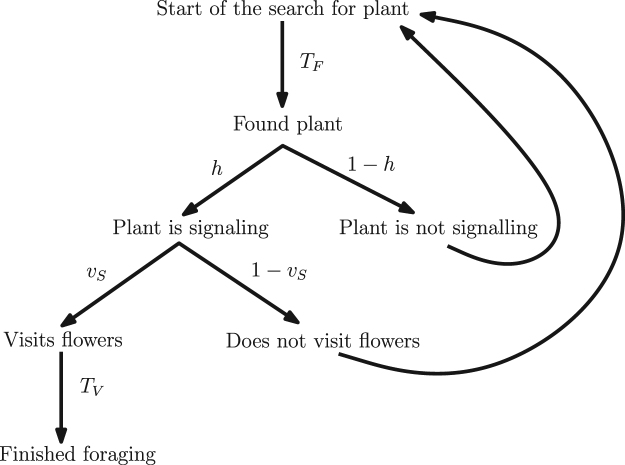
Determining the foraging rate for the pollinators if they visit only signaling high-yield plants. It takes time *T*_*F*_ to find a plant. With probability 1 − *h*, the plant is not high-yield and the pollinator keeps searching for the plant. If the plant is signaling, the pollinator may still skip it with the probability $1-{v}_{S}$ (and then keeps searching for the plant). With probability *v*_*S*_, the pollinator visits the plant and spends time *T*_*V*_ foraging on flowers.

We are going to determine how long, on average, does it take the pollinator to receive a reward from a plant, assuming the pollinator visits only (signalling) high-yield plants. Let *T* denote the expected value of such a time. It takes time *T*_*F*_ for the pollinator to find a plant. The pollinator will then visit the plant only if the plant is signaling (which happens with probability *h* as the only signaling plants are high-yield) and only if it decides to visit (with probability *v*_*S*_), i.e. in total with probability *hv*_*s*_. In all other cases, it continues to search. When the pollinator eventually visits the plant, it extracts the reward in time *T*_*V*_. This is shown in Fig. 2.

Thus, $T={T}_{F}+h{v}_{S}{T}_{V}+(1-h{v}_{S})T$ and consequently, $T={T}_{V}+\frac{{T}_{F}}{h{v}_{S}}$.

The long-term feeding rate, i.e. the amount of reward collected per unit of time, of such a pollinator is then $\frac{{V}_{H}}{T}$.

### Learning to skip low-yield plants

Skipping a visit of a low-yield (non-signalling) plant is beneficial for the pollinator if the instantaneous feeding rate $\frac{{V}_{L}}{{T}_{V}}$ (should the pollinator decide to visit the plant) is smaller than the long-term feeding rate $\frac{{V}_{H}}{T}$. After some algebra, the condition $\frac{{V}_{H}}{T} > \frac{{V}_{L}}{{T}_{V}}$, yields

condition 1 $$\frac{{V}_{H}}{{V}_{L}} > 1+\frac{1}{h{v}_{S}}\,\frac{{T}_{F}}{{T}_{V}}.$$

Under (condition 1), pollinators will learn to skip low-yield plants, i.e. *v*_*N*_ will tend to *v*_*min*_ Since high-yield plants provide the best feeding rate possible, pollinators automatically learn to increase *v*_*S*_ and thus *v*_*S*_ tends to *v*_*max*_ Consequently, (condition 1) eventually becomes

condition 1′ $$\frac{{V}_{H}}{{V}_{L}} > 1+\frac{1}{h{v}_{max}}\,\frac{{T}_{F}}{{T}_{V}}.$$

### Conditions for benefits of signalling

The population where high-yield plants are signaling plants and low-yield are not will be stable if (a) a rare non-signalling high-yield plant will do worse than prevalent signaling high-yield plants, and (b) a rare signaling low-yield plant will do worse than prevalent low-yield plants. Part (a) will be satisfied if

condition 2 $$R({v}_{{\rm{\max }}})-{C}_{H} > R({v}_{min}).$$

Part (b) will be satisfied if

condition 3 $$R({v}_{{\rm{\min }}}) > R({v}_{max})-{C}_{L}.$$

Combining (conditions 2 and 3) yields a necessary condition *C*_*L*_ > *C*_*H*_, i.e. it must be costlier for a low-yield plant to signal than it is for a high-yield plant.

For the illustration, if a reward function ${R}_{multi}(v)={R}_{0}vP$ is adopted, (conditions 2 and 3) become

$${C}_{H} < {R}_{0}P\,({v}_{max}-{v}_{min})\,{\rm{and}}\,{C}_{L} > {R}_{0}P\,({v}_{max}-{v}_{min})$$

giving some concrete and testable predictions about costs of the signals. At the same time, if a reward function ${R}_{single}(v)={R}_{0}(1-{(1-v)}^{P})$ is adopted and we assume large *P*, we get that ${R}_{single}(v)\approx {R}_{0}$ for most values of *v* > 0 and thus it is possible that neither (condition 2) nor (condition 3) can be satisfied.

#### Other equilibria

There is also so called pooling equilibrium where no plant signals and where pollinators visit plants randomly. In fact, such an equilibrium exists and is evolutionarily stable under all conditions because when only a very small invading fraction of the plants signal, pollinators will not be able to learn the meaning of the signal and thus they will visit all plants with the same probability (and it is therefore not beneficial to pay a cost to signal).

Situation when all plants signal is not evolutionarily stable. If only a small fraction of low-yield plant stopped signalling, the pollinators would not be able to learn the meaning of the absence of the signal, so they would still visit all plants with the same probability. Yet, the non-signalling low-yield plants would not pay the signalling cost, i.e. they would do better than the signalling low-yield plants and thus they can successfully invade.

Situation when only low-yield plants signal is not evolutionary stable either. If a small fraction of the low-yield plants stopped signalling, the pollinators would visit them more often (as often as they visit high-yield plants), yet the non-signalling low-yield plants would not have to pay the signalling cost and so they would do better than the signalling low-yield plants.

Because the pooling equilibrium exists independently of the above conditions 1, 2 and 3, plants may do better (or worse) in the pooling equilibrium than under honest signalling. Also, pooling equilibrium and honest signalling equilibrium may coexists. Our model cannot predict which of the possible two is more likely to evolve but see for example Bergstrom and Lachmann^35–37^ and Zollman *et al*.^38^.

## Discussion

We presented a simple yet general model based on the repeated two-type Spence signalling game between plants and pollinators. Our model is similar (but not identical) to the Sir Phillip-Sydney game^18^. Also, the analysis of the Sir Phillip-Sydney game using ESS and Evolutionary game theory as shown for example in Huttegger & Zollman^39^ or Catteeuw *et al*.^40^ is similar to our analysis and authors arrive to similar observations (such as honest signalling must be costly).

In our model pollinators can learn and adjust their preference to visit signaling and non-signalling plants. The model was general in a sense that we did not use any explicit formulas for plants’ benefits nor any explicit learning or reputation model (see e.g. Silk *et al*.^41^; Catteeuw *et al*.^42^; Rich and Zollman^43^). Our model gave us three conditions under that honest signalling is evolutionary stable equilibrium.

(Condition 1) or its equivalent (condition 1′) means that for the honest signaling to be stable, the relative difference between pollinator’s gains from the high-yield versus low-yield plants, $\frac{{V}_{H}-{V}_{L}}{{V}_{L}}$, should be quite large. If the difference is large, pollinators learn to skip low-yield plants in favor of searching for high-yield plants. Explicitly, the relative gain difference should be larger than $\frac{{T}_{F}}{{T}_{V}}$ (a time to find a plant measured in units of time to visit a plant) times $\frac{1}{h{v}_{S}}$ (which corresponds to the reciprocal value of a frequency to visit a high-yield signaling plant). In particular, high-yield plants should be abundant in the population (*h* should be large), and it should not be difficult to find a plant (*T*_*F*_ should not be too large).

Condition 2 means that the benefits of attracting more pollinators outweigh the associated cost of signaling for a high-yield plant.

It is expected that investment in attracting the pollinators increases the fitness of the plants (via increasing dispersal and receipt of pollen) at least as much as the cost decreases it as plants may have less resources to produce seeds and/or pollen^44^. The cost-benefit analysis of the signaling in *D. scandens* has been done by Pélabon *et al*.^44^. In *D. scandens*, plants provide resins secreted by a gland-like structure in the inflorescence as pollinator rewards, which could be honestly advertised through the size of resin-producing glands. The results of the experimental manipulation of resin production suggested that resin secretion has no cost in terms of seed production^44^. However, little is known about the costs of floral signals and more studies are needed to evaluate the environmental effects on production and maintenance of the reward costs. The synthesis of floral scent compound could be at the expense of reducing the synthesis of other amino acid-based metabolites^14^.

Condition 3 is satisfied if the cost of dishonest signaling is so high that attracting more pollinators is not beneficial. It may so for several reasons

- $R({v}_{min})$ may already be relatively close to $R({v}_{max})$ – potentially if the low-yield plants can be pollinated by wind etc. and do not really need pollinators so much. This may also be the case of pollinators’ abundance if the reward function for plants behave like *R*_*single*_, i.e. if it is close to constant when pollinators are abundant.
- *C*_*L*_ is prohibitively large for low-yield plants. The production of signal and reward is costly and resource limited^14^. The plants with a low resource allocation to flowers can’t afford to produce the signals and large rewards.

Note that Leshowitz (MA thesis 2017 at UNCG) built a very similar model but did not consider the times it takes to extract the reward and times it takes to find a plant^45^. Without considering the times, honest signaling was not stable equilibrium even if (conditions 2 and 3) were satisfied. Inclusion of the times and (condition 1) was the crucial part of this model that allowed for the honest signaling to be stable. The condition 1 indicated that the establishment of honest floral signals could be determined by the frequency of high-yield plants in the population and the encounter rates between pollinators and plants with different qualities. The encounter rates could be dependent on the density of plants. So the frequency- and density-dependent interaction with pollinators could determine the establishment of honest signalling.

Nevertheless, our model is simple and does not account for potential complexities. We assumed only two possible yields from plants, high and low, rather than a whole spectrum of yields (Spence signalling game can be extended for situation where plant quality is continuous, see e.g. Grafen 1990^16^). We assumed that high-yield and low-yield plants are otherwise same or very similar (for example their rewards functions match). We also assumed only one kind of pollinator. We did not consider any dishonesty from the pollinator side (i.e. when the pollinator went for a reward, it provided the pollination benefit for the plant rather than somehow cheating the plant out of it). Another complication not considered by our model is that the plant with reward depleted by recent visits could send a dishonest signal if its signal is still retained. The pollinator, which subsequently visits this plant, need to be tolerant of flowers with variable return, reducing selection for honest signals^15^. The tolerances of pollinators are unlikely to impose selection on cost-enforced signalling^46^. Meanwhile, we also assumed that the pollinators have enough time to learn. But there is the cost of time associated with learning. For example, the time required to discriminate the nectar or nectarless flowers could influence the producing the proportion of nectarless flowers in plants^47^. The existence of other equilibria could support an existence of the continuum from dishonest to honest signalling in plant-pollinator interaction. Meanwhile, it also indicated that pollinators’ learning ability and foraging strategies could determine the effectiveness for resolving partner choice by signalling mechanism.

We also assumed that the signalling itself does not attract pollinators more to the plants for further inspection. So, the frequencies of different types of plants determine the encounter rates. This assumption may not be realistic as the visual and olfactory signals can attract pollinators, which in turn means that real encounter rates do not match encounter rates at random. Nevertheless, our model can be modified if needed to account for the shortcomings mentioned above. For example, if signaling is in the form of scent and the scent makes plants easier to find for pollinators, the true meaning of *h* in our model will be the actual relative frequency pollinators encounter high-yield plants rather than the relative frequency of high-yield plants in the population. As such *h* can still be experimentally measured although the experiment set up will be different than if *h* means just the frequency of high-yield plants.

### Testable predictions

- Honest signalling does not develop if pollinators are too common (plants get eventually visited and thus get their rewards even if they do not advertise).
- It could be expected that when pollinators are scarce, plants could invest greater allocation to attractiveness, which could be contributed to the establishment of honest signaling. In *Alkanna orientalis*, plants can increase resources allocation for floral traits and nectar to attracting pollinators in response to the spatial associated with fewer pollinating visits^48^. So, the honest signalling may be more common in pollinator-limited plants. Recently, some evidences showed that there is reduction of pollinator richness and density at a global scale^49^. Thomann *et al*.^50^ propose that plants could increase reinforced interactions with pollinators by increasing investment in attraction to deal with current pollinator decline^50^. This could provide the chance to test the prediction of our model.
- Honest signaling does not develop when there are not many plants for the pollinators (as it takes a pollinator too much time to find another plant, so it prefers to get its reward even from a low-yield plant).
- The density-dependent relationship between plants and pollinators is important for the establishment of honest signalling. In *Clarkia concinna*, low plant density patches can limit pollinator attraction, resulting in reproductive failure^51^. The high plant density can increase the encounter rate with pollinators. Under this situation, pollinators can evaluate the signal-reward relationship and prefer plants providing high returns. But when plants were scarce, pollinators may not discriminate between flowers with different reward amounts^52^. So the establishment of honest signalling is dependent on the positive density-dependent interaction between plants and pollinators. We expect that the signal-reward relationship can vary with plant density.
- Even when plants already signal honestly, if there is a sudden decrease of plant frequency, pollinators learn to visit even the low-yield and non-signalling plant (because it takes too long to find another plant).
- Meanwhile, the establishment of honest signalling is also dependent on frequency-dependent interaction between plants and pollinators. Pollinators foraged at high-yield plants at greater visiting rates when high-yield plants were at high frequency, and at lower visiting rates when they were rare^53^. So, at low frequencies of high-yield plants, pollinators will visit both types of plants indiscriminately. As the frequency of high-yield plants increases, pollinators are expected to avoid low-yield plants. Positive frequency-dependent pollination efficiency could promote the honest signalling. And negatively frequency-dependent selection mediated by pollinators could maintain the dishonest signals in plants^53^.
